# Single-Particle Discrimination of Retroviruses from Extracellular Vesicles by Nanoscale Flow Cytometry

**DOI:** 10.1038/s41598-017-18227-8

**Published:** 2017-12-19

**Authors:** Vera A. Tang, Tyler M. Renner, Anna K. Fritzsche, Dylan Burger, Marc-André Langlois

**Affiliations:** 1 University of Ottawa Flow Cytometry and Virometry Core Facility, Ottawa, Canada; 2 Department of Biochemistry, Microbiology and Immunology, Faculty of Medicine, University of Ottawa, Ottawa, Canada; 3 Department of Cellular and Molecular Medicine, Faculty of Medicine, University of Ottawa, Ottawa, Canada; 4 uOttawa Center for Infection, Immunity and Inflammation, Ottawa, Canada

## Abstract

Retroviruses and small EVs overlap in size, buoyant densities, refractive indices and share many cell-derived surface markers making them virtually indistinguishable by standard biochemical methods. This poses a significant challenge when purifying retroviruses for downstream analyses or for phenotypic characterization studies of markers on individual virions given that EVs are a major contaminant of retroviral preparations. Nanoscale flow cytometry (NFC), also called flow virometry, is an adaptation of flow cytometry technology for the analysis of individual nanoparticles such as extracellular vesicles (EVs) and retroviruses. In this study we systematically optimized NFC parameters for the detection of retroviral particles in the range of 115–130 nm, including viral production, sample labeling, laser power and voltage settings. By using the retroviral envelope glycoprotein as a selection marker, and evaluating a number of fluorescent dyes and labeling methods, we demonstrate that it is possible to confidently distinguish retroviruses from small EVs by NFC. Our findings make it now possible to individually phenotype genetically modified retroviral particles that express a fluorescent envelope glycoprotein without removing EV contaminants from the sample.

## Introduction

Retroviruses, such as the human immunodeficiency virus (HIV), are enveloped RNA viruses that range between 90–150 nm in diameter, depending on the species^1–3^. When nascent virions egress from infected cells, they bear contents of the cytosol (e.g., proteins, mRNAs, miRNAs), as well as a portion of the cell membrane embedded with surface receptors to form the viral envelope^4–6^. The phenotypic analysis of host-derived markers on the surface of individual viruses is of considerable interest, as it can provide information on the identity of the specific cell types that are infected in a host. However, a major hurdle in purifying retroviruses for single-particle characterization studies is the removal of EVs that are concomitantly released by the cells^7–11^. EV is a broad term that describes all particles with a membrane bilayer released from cells; these can include exosomes, microvesicles, and apoptotic vesicles^12–16^. Small EVs, that are in the size range of retroviruses constitute a major contaminant of virus preparations as they are biochemically and biophysically similar to retroviruses in terms of their refractive indices, buoyant densities, and surface markers^5,7,17–19^. Additionally, EVs can also package retroviral proteins and RNAs that further complicate discrimination^20–24^.

Nanoscale flow cytometry (NFC), also called flow virometry, is a new and powerful tool in the field of virology that enables the phenotypic analysis of the markers at the surface of individual virions^11,25–32^. Virus populations can now be profiled and sorted in multi-parameter analyses, much in the same way as cells^25,27,30,32,33^. However, NFC analysis with current instrumentation can be challenging due to the fact that these particles are at the limit of detection for flow cytometers. The research community is working towards the standardization of flow cytometer requirements, as well as acquisition settings and labeling procedures for NFC analysis, but there is yet to be a consensus^34–36^. To complicate matters, instruments of different make will have different optics, fluidics, electronics, detectors, and software. Even using the same instrument model, variations in sensitivity and resolution are common given that the instruments are operating at the threshold of their physical limits of detection. This can be much lower than the published manufacturer’s specifications, but the onus falls on individual users to achieve this. Despite these caveats, several groups are currently using conventional flow cytometers originally designed for cells to analyze nanoparticles in the 90–150 nm size range^11,25–34,36–40^. Common instrument hardware additions for small particle detection include lasers with higher power and the use of photomultiplier tubes (PMTs) instead of photodiodes for forward scatter detection. Most commercial flow cytometers that claim small particle detection capabilities have a lower size limit in the 100 to 300 nm range based on detection of beads. However, there is currently no consensus in the field as to the minimum laser power required for the consistent detection and resolution of nanoparticles. While conventional flow cytometers clearly have the capacity to detect nanoparticles to varying degrees of sensitivity, standardization of instrument settings and sample acquisition procedures is necessary for cross-laboratory data validation and reproducibility.

Here we undertook a systematic approach to analyze viruses by NFC using a special order research product (SORP) BD LSR Fortessa flow cytometer. The model virus for our study is the Moloney murine leukemia virus (MLV), as it is a well-studied and characterized retrovirus that is non-infectious for humans. We identified the optimal settings for laser power and voltage to provide maximum particle enumeration and resolution, as well as sample dilutions and flow rates to minimize coincidence. We then compared data acquired using either fluorescence or side scattered light (SSC) as the threshold for detection. Finally, we compared various dyes and staining methods that can be used to discriminate MLVs from the EV contaminants.

## Results

### Analysis of single virions by NFC

**Figure 1:**
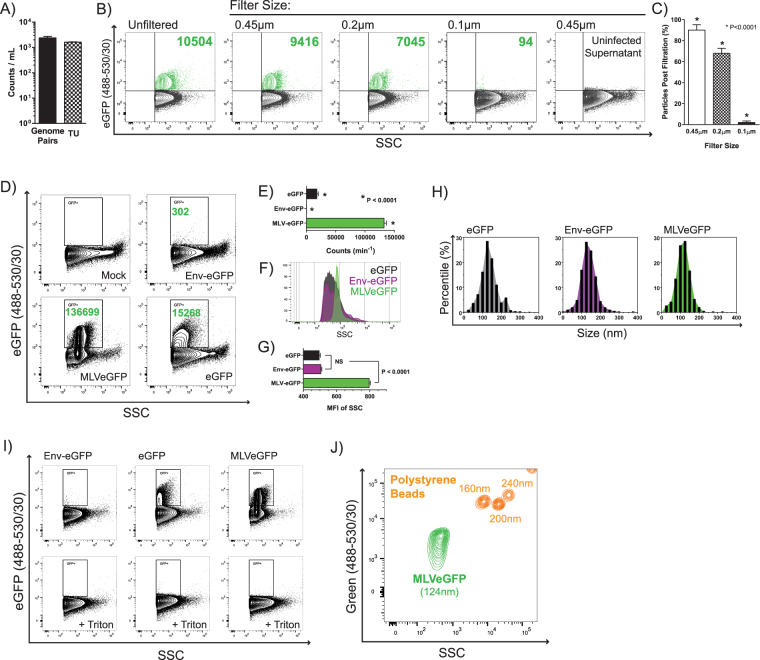
Analysis of MLVeGFP virions by NFC. (**A**) MLVeGFP viral particles from chronically infected NIH 3T3 cell supernatants were filtered through 0.45 μm pore-sized PES cartridge filters and titered on NIH 3T3 cells for TU analysis. The same supernatant was analyzed by ddPCR for genome analysis. Both of these values were related back to the input volume of supernatant. (**B)** MLVeGFP from chronically infected NIH 3T3 cell supernatants were filtered through 0.45 μm, 0.2 μm and 0.1 μm pore-sized PES cartridge filters. eGFP+ particles are depicted in green and background noise and/or non-fluorescent particles in black. The number of eGFP+ events in the top right quadrant gate are indicated in green. (**C**) eGFP+ particle count post filtration as a percentage of the total eGFP+ particles in the unfiltered virus preparation. Statistical significance was calculated by 1-way ANOVA. (**D**) 293 T cells were transfected with expression plasmids for eGFP or Env-eGFP, or with the MLVeGFP expression plasmid. eGFP+ particles were analyzed by NFC as above. Transfection efficiency was approximately 75% in all conditions (data not shown). (**E**) eGFP+ particles from three independent transfections experiments as described in C, were tabulated. Average particle counts with standard deviation (S.D.) are presented. Statistical significance was calculated by 1-way ANOVA. (**F**) SSC intensity comparison of eGFP+ eGFP+ EVs and virus shows that MLVeGFP displays a more homogenous particle population that scatters light more intensely. (**G**) Quantification of SSC intensities (MFI) from three independent experiments with S.D., as described in F. P values were calculated by Student’s t-test. (**H**) Representative size profiles of particles released from transfected 293 T cells expressing eGFP, Env-eGFP, and MLVeGFP. Samples were analyzed by nanoparticle tracking analysis (NTA). Results are displayed as the percentage of particles within 25 nm segments. (**I**) eGFP expression in EVs (eGFP and Env-eGFP) and MLVeGFP virus was abrogated by treatment with 0.05% Triton X-100. (**J**) Comparison of polystyrene green fluorescent beads and MLVeGFP on SSC vs. Fluorescence (Green 488–530/30).

For this study, we used the Moloney MLV expressing a chimeric envelope-eGFP surface glycoprotein (MLVeGFP). Moloney MLV is an enveloped virus that is nearly spherical with a mean diameter of 124 nm as measured by cryo-electron microscopy^2^. It is estimated that there are approximately 100 envelope glycoprotein spikes per MLV virion, which is nearly an order of magnitude more than the 7–14 gp120 spikes found at the surface of HIV-1^41–43^. We have shown previously that enveloped viruses can form aggregates when subject to centrifugation and repeat freeze thaw^32^. For this reason, and to reduce other contaminants and purification artefacts, virus-containing supernatant from chronically infected NIH 3T3 cells cultured in 0.1 µm filtered, serum and phenol red-free media was analyzed directly by NFC. We used a sample acquisition time of 60 seconds in all experiments of this study. The production of non-infectious particles was minimal with MLVeGFP, as there was a direct correlation between genome counts and infectious units (Fig. 1A). Similar high-efficiency viral genomic RNA (gRNA) packaging was previously reported for HIV-1^44^.

To ensure that cellular debris is removed and that virus was present in single-particle suspensions, we used microfiltration (Fig. 1B). Virus-containing supernatants were passed through 0.45, 0.2 and 0.1 μm filters, diluted 100-fold in 0.1 μm-filtered PBS, and analyzed by NFC. Filtration through a filter with a 0.45 μm pore size removed approximately 10% of total eGFP+ particles, while filtering through a 0.2 μm cartridge removed 30% of eGFP particles (Fig. 1B and C). Passing the sample through a 0.1 μm filter removed nearly all eGFP particles, as expected, and displayed an overall residual particle profile similar to 0.45 μm-filtered uninfected cell supernatant (Fig. 1B).

Because EVs can acquire viral proteins and nucleic acids when they are released from cells, we next sought to determine what proportion of all eGFP+ particles constitute virus. To measure the degree of MLV envelope-eGFP (Env-eGFP) incorporation into EVs, we cloned the coding sequence of Env-eGFP into an expression vector and transfected it into 293 T cells. As a control for this experiment, MLVeGFP was produced by transfection as well. Transfection efficiencies for the eGFP and Env-eGFP plasmids were consistently between 45% and 55%, the transfection efficiency of the plasmid coding for MLVeGFP was around 8–10%. All samples were passed through a 0.45 μm filter and diluted 100-fold in PBS prior to NFC analysis. Here we found that eGFP+ particles were 450-fold more abundant in the virus-containing cell supernatant than in supernatant containing EVs released from Env-eGFP transfected cells (Fig. 1D and E). Interestingly, when the cells were transfected with an expression plasmid coding only for eGFP, the number of eGFP+ particles was higher, representing 10% of the total eGFP+ count of the viral sample. These results indicate that by NFC analysis, Env-eGFP appears to be almost exclusively present in the culture supernatant when the virus is present. However, very low levels of particles clearly do express Env-eGFP when virus is absent. Particles released in cells transfected with eGFP or Env-eGFP displayed different side scatter (SSC) and fluorescence intensities than particles in the MLVeGFP containing supernatant (Fig. 1F and G). Viral particles had a higher SSC mean fluorescent intensity (MFI) and a more homogenous distribution, despite having a similar size distribution profile as measured by nanoparticle tracking analysis (NTA) (Fig. 1H).

To confirm that detected eGFP+ particles are indeed EVs and viruses, and not protein aggregates, all samples were subjected to treatment with Triton X-100 detergent, which is a effective way to dissolve EVs and strip the retroviral envelope leaving capsids intact (Fig. 1I)^45,46^. The eGFP fluorescent signal was lost in all samples. Therefore, the data presented in this figure support that most of the eGFP+ particles produced from MLVeGFP infected cells appear to be virus.

Finally, to emphasize the disparity of refractive indices and fluorescence between synthetic bead size standards and biological particles, we compared MLVeGFP with green fluorescent polystyrene bead populations that ranged from 160–240 nm (Fig. 1J). Although MLVeGFP is only 40 nm smaller in diameter than the 160 nm beads, it displayed a SSC MFI that was approximately ten-fold lower. In comparison, the difference in SSC intensity between the 160 nm and 240 nm bead populations was only about 3-fold. This comparison serves to demonstrate that a population of biological particles of similar size to a polystyrene bead population tends to display a much lower SSC MFI.

### Impact of thresholding and sample dilution on electronic aborts and event counts

In order to have analyzed eGFP+ particles in the previous section, several NFC parameters had first needed to be optimized which include voltage, laser power, flow rates and sample dilutions. The following sections describe in detail how these settings were determined and also how they affect data acquisition.

Coincidence occurs when two or more particles are interrogated simultaneously^36–38,47,48^. Flow cytometers are specifically designed to create a stream of single cells that are individually analyzed by the instrument. Because nanoparticles are much smaller than cells, it is more likely that several particles are coincidentally interrogated at the same time if the sample is too concentrated. Each event where the signal is above the designated threshold is registered as a voltage pulse with a height, width, and area parameter. The height is the intensity of the pulse, while the width is the time of flight of the particle during laser interrogation. Area is the integrated value under the voltage pulse, which represents the intensity of the signal over time as calculated using the height and width values. In BD FACSDiva, each pulse is assigned a windows extension, which is a specific measure of time at the beginning and end of each voltage pulse. If the windows extension of two events overlap, as in the case of coincident events, the signals will not be processed and will instead be aborted. This is what is called an electronic abort^49^. Therefore, if a very concentrated sample is analyzed, a large number of electronic aborts will occur and data events will be discarded. This constitutes a major concern when analyzing nanoparticles by NFC.

Additionally, since the signal generated by biological nanoparticles is very dim (Fig. 1J), the threshold for detection is set at or near the lower limit. Low threshold values translate into reduced stringency of what is considered an event and this results in an increased likelihood of coincidence and background noise. Thresholding off of SSC will result in more particles analyzed by virtue of the fact that all particles, including any sample contaminants (i.e. PBS crystals, protein aggregates), will have size. On the other hand, a fluorescence threshold will generally be set at a slightly higher value to visualize only the fluorescently labeled particles of interest. This theoretically should result in fewer electronic aborts when comparing identical samples since there will be less total events processed. However, the compromise is losing information about total particles in the sample (i.e., very dim or unlabeled particles). Here we have systematically compared the effects of sample dilutions and flow rates on both SSC and fluorescence thresholding on total counts for our particles of interest.

**Figure 2:**
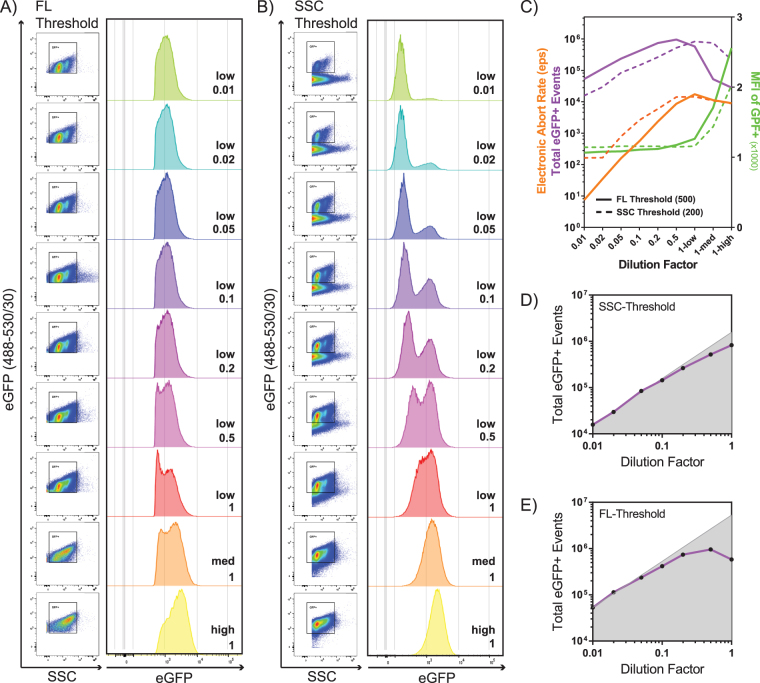
Effect of fluorescence and SSC thresholding on particle counts and electronic aborts. Effects of sample dilutions and flow rates on data acquisition. (**A**) MLVeGFP was analysed with the event threshold set at a fluorescence intensity of 500 on green fluorescence channel (488–530/30) (FL-Threshold) and (**B**), SSC from the 488 nm laser at a fluorescence intensity of 200 (SSC-Threshold). Serial dilutions were performed on 0.45 μm-filtered cell supernatants: undiluted: 1; 1/2: 0.5; 1/5: 0.2; 1/10: 0.1; 1/20: 0.05; 1/50: 0.02; 1/100: 0.01. All diluted samples were analyzed with the sample flow rate set to low. Undiluted samples were analyzed with flow rates set to low, medium (med) and high. (**C**) Electronic aborts, Total eGFP events and median fluorescence intensity (MFI) of eGFP+ particles were plotted as function of the dilution factor and flow rate. SSC-threshold (dotted line) and FL-threshold (solid line). Total counts of eGFP+ particles were plotted for SSC-thresholding (**D**), and FL-thresholding (**E**), against a linear regression plot of predicted events (grey area) based on counts measured for the 1/100 dilution for each thresholding condition. Data is representative of two separate experiments.

Serial dilutions of a single sample of 0.45 μm-filtered supernatant containing MLVeGFP produced from chronically infected NIH 3T3 cells was analyzed using the *low* sample flow rate setting. The undiluted sample was further analyzed on *medium* (med) and *high* settings to further exaggerate and emphasize the effects of coincidence and electronic aborts. The average virus titer in the undiluted filtered supernatant remained constant throughout replicate experiments at approximately 1.5–3 × 10^6^ transducing units (TU)/mL. The samples were analyzed using fluorescence thresholding (Fig. 2A) or SSC thresholding (Fig. 2B), and the electronic abort rates were manually recorded. The optimal sample concentration and flow rate are where signal intensities stabilize and event rates linearly correlate with changes in sample concentration^36–38,47,48^. Sample acquisition data was plotted to display electronic aborts, total events and median fluorescence intensity as a function of sample dilution and increasing flow rates for the undiluted sample (Fig. 2C). Total events acquired using SSC (Fig. 2D) and fluorescence (Fig. 2E) thresholding were separately plotted against a linear regression curve in order to emphasize the dilutions in the linear range. Linear regression of the predicted number of eGFP+ events (shaded area) was extrapolated using the eGFP+ counts from the most dilute sample for each thresholding condition. Our data shows that electronic aborts steeply rise as the samples become more concentrated. Consistent with coincidence, an increase in electronic aborts correlates with a rise in mean fluorescence intensity (MFI) of particles (Fig. 2C). Surprisingly, the increase in MFI is not seen until the undiluted sample is run at *medium* or *high* sample pressures. Total and eGFP particle counts are inversely proportional to the dilution factor of the sample when in the range of 0.01 to 0.05 (Fig. 2D and E). Contrary to what would be expected, particle counts appear to be slightly lower using SSC compared to fluorescence thresholding by a factor of 3. This is likely due to the higher rate of electronic aborts using SSC thresholding, even at a dilution of 0.01 (Fig. 2C). Taken together, the data shows that a stable MFI alone does not necessarily indicate that the sample is running at an optimum event rate. While there is a wide window of dilutions that provide constant MFI values, event count analysis provides a more accurate way of determining optimal flow rates and sample dilutions.

### Effects of voltage and laser power settings on particle detection, resolution and electronic aborts

**Figure 3:**
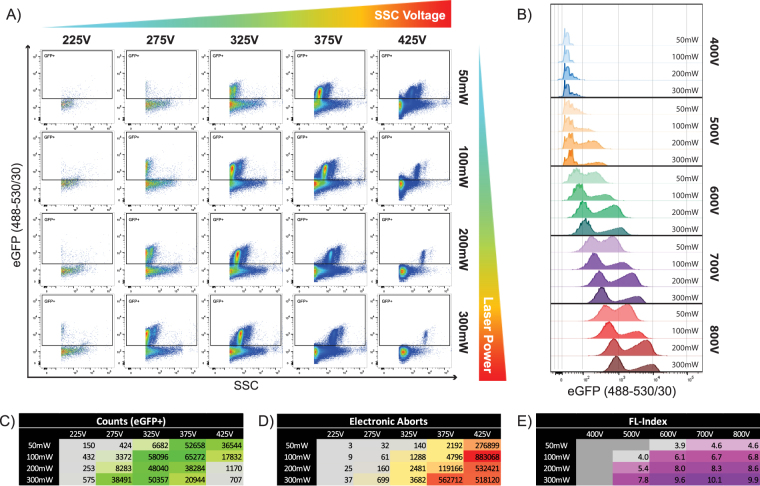
Effects of laser power and PMT voltages on fluorescence and SSC. MLVeGFP was analysed with systematic adjustments of voltage for detection of green fluorescence and SSC off the 488 nm laser. (**A**) Increments of 50 V were made on SSC off the 488 nm laser from 225 V to 425 V while maintaining a consistent MFI for the eGFP+ population on the green fluorescence channel (488nm-530/30), while simultaneously increasing laser power from 50 to 300 mW. Threshold was set at SSC fluorescence intensity 200. (**B**) The same voltage and laser power adjustments were made on the green fluorescence channel while maintaining constant the MFI in SSC of the eGFP+ population. (**C**) Tabulation of the number of eGFP+ events in gates set in A. (**D**) Tabulation of the number of electronic aborts generated during the acquisition of each condition described in A. (**E**) Fluorescence Index calculated for each condition portrayed in B. FL-index = (MFI_eGFP+_ − MFI_eGFP−_)/SD_eGFP−_. Data is representative of three separate experiments.

Here we systematically measured the effect of increasing voltage and laser power on our virus preparation (Fig. 3A). We began with the 488 nm laser at full power (300 mW) and increased the SSC voltages in increments of 50 V, and then gradually reduced laser power all the way while keeping eGFP MFI of our population of interest constant. In a subsequent acquisition set, we also increased green fluorescence (488–530/30) detector voltage in increments of 100 V (while keeping the SSC MFI constant) to determine the range of settings at which eGFP+ particles are detected (Fig. 3B). Threshold was set on SSC off the 488 nm laser at the lowest value permitted by our acquisition software, which is 200 relative fluorescence intensity units. We chose to threshold off of SSC to include detection of all fluorescent and non-fluorescent events. Because acquisition is carried out on the same sample, changes will reflect the effects of the settings. When voltages were increased on SSC, the noise and event rate also increased to a certain limit (Fig. 3C), which correlated with increased electronic aborts (Fig. 3D). The increased electronic abort rate at higher SSC voltage settings resulted in a diminished number of total eGFP+ particles being acquired.

When comparing data collected for a given SSC voltage setting, increase in laser power resulted in an increase in the separation of the MFI of SSC signal intensities between the eGFP+ and eGFP− populations (Fig. 3A). This is most evident in the voltage range between 325 to 425 V for SSC, where the eGFP+ population is above threshold on SSC. Furthermore, peak particle counts was reached at a lower voltage as laser power increases (Fig. 3C). This tracked with electronic aborts, which were at their maximum when laser power and PMT voltages were at their highest settings (Fig. 3D).

When laser power and voltage on the green fluorescence channel were increased, it was evident that signal intensities of both the eGFP+ and eGFP− populations were also increased (Fig. 3B). To determine if the increase of laser power resulted in an increase in separation of the positive and negative populations in the green fluorescence channel, a fluorescence index (FL-index) value was calculated. This value is based on the difference in the MFI of the eGFP+ and eGFP− populations divided by the standard deviation of the eGFP− population (Fig. 3E). Samples where there was no clear resolution of an eGFP+ population were omitted (grey boxes). When comparing samples acquired at the same voltage, higher laser power provided improved resolution of the fluorescence signal (defined by the separation of the positive population from the negative) as represented by a higher FL-index value (Fig. 3E). These results clearly indicate that our virus can be detected over a wide range of voltage and laser power settings, however greater laser power more noticeably improves fluorescence and scatter resolution.

### Discrimination of retroviruses from extracellular vesicles using fluorescent dyes

To date, several groups have demonstrated the ability to label EVs and viruses through the use of commercially available dyes^26,32,33,36,50^. Dyes that target different cellular components, such as proteins, lipids and nucleic acids, have been proven effective to stain EVs and viruses in flow cytometry applications. However it has yet to be demonstrated that these methods can be used to discriminate between viruses and EVs.

To identify both virus and EV populations in our infected cell supernatants, we used fluorescent dyes that target cellular membrane components. Dyes were selected over the use of antibodies due to their ability to stain particles based on their biochemical constituents. Dyes are also not subject to some of the limitations of antibodies such as low surface antigen expression, epitope heterogeneity, and particle aggregation^32^. There is currently a large number of commercially available dyes that target different molecules and cellular components. Using supernatants containing MLVeGFP, we first tested lipid dyes DiD, DiI, FM4-64, fluorescent sphingolipid (Ceramide-BODIPY TR) and fluorescent phospholipid (DHPE-Rhodamine). Several groups have published methods to directly label virus with dye^51–54^, however these studies did not account for EV contamination in virus sample preparations. In our system, virus particles are distinguished from EVs by the surface expression of Env-eGFP. EVs are largely devoid of Env-eGFP on their surface (Fig. 1D), and therefore will only emit fluorescence at the wavelength of the dye.

**Figure 4:**
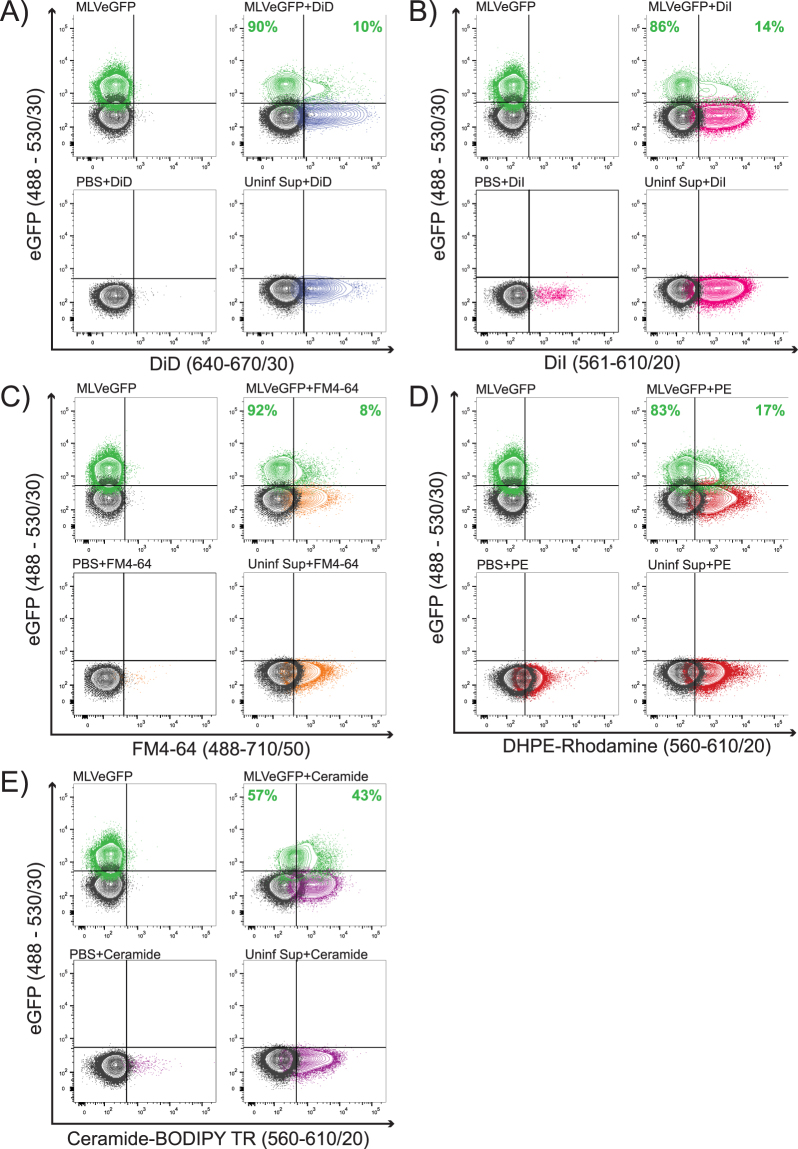
Direct staining of virus. MLVeGFP viruses from chronically infected NIH 3T3 cell supernatants were labeled with (**A**) DiD, (**B**) DiI, (**C**) FM4–64, (**D**) Rhodamine-1,2-DihexadecanoylPhosphatidylethanolamine (DHPE-Rhodamine), and (**E**) Ceramide-BODIPY TR. Samples were then analyzed by NFC without purification. eGFP+ particles are depicted in green, background noise or non-fluorescent particles in black, dye-labeled particles in color (other than green). The distribution of total dye-labeled and unlabeled eGFP+ particles is indicated in green. Included in each panel: unstained virus (MLVeGFP), stained virus (MLVeGFP+ dye), dye alone (PBS + dye), and uninfected supernatant with dye that serves as an EV-only sample (Uninf Sup + dye).

For all the dyes tested, the direct sample staining approach discriminated virus from EVs by strongly labeling EVs while not detectably labeling virus (Fig. 4). Ceramide-BODIPY TR was the only dye that labeled the virus population at a low level, which was not completely resolved from background (Fig. 4E). Since excess dye was not washed away in this method, stained particles were detected in the PBS controls for DiI, DHPE-rhodamine and Ceramide-BODIPY TR, but not with DiD or FM4-64. This may reflect insoluble dye aggregates or micelle formation as evident with other lipophilic dyes^50^. In this method of direct labeling, we show that the majority of viruses did not uptake dye, except for a small fraction of dual labeled particles that consistently represented between 8–17% of the virus population.

**Figure 5:**
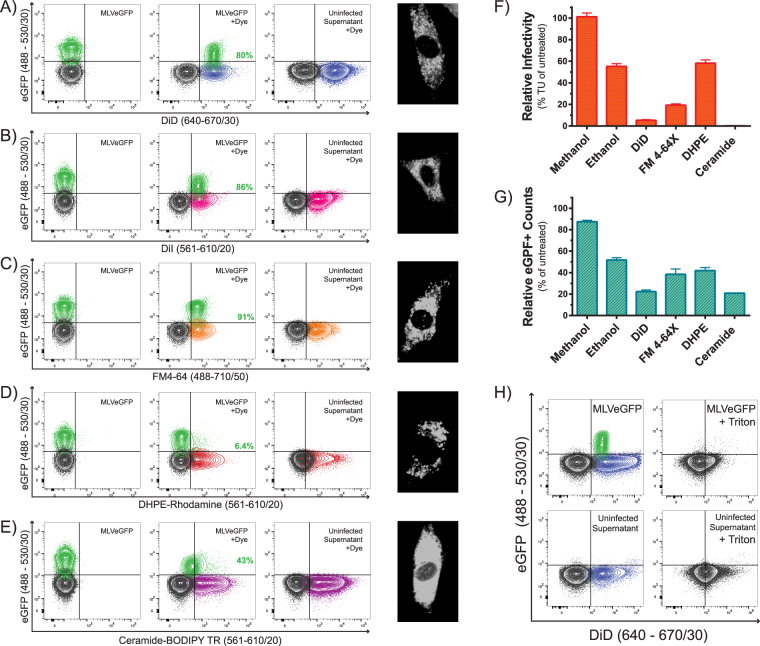
Indirect staining of virus. Supernatants of chronically infected and uninfected cells labeled with (**A**) DiD, (**B**) DiI, (**C**) FM4-64, (**D**) DHPE-Rhodamine, and (**E**) Ceramide-BODIPY TR were analyzed by NFC. (**F**) Relative virus infectivity (transducing units (TU) per mL) and (**G**) counts of eGFP+ particles released from the supernatants of infected cells labeled with the different dyes. Infectivity and counts are presented as the percentage of total number of eGFP+ particles released from unstained virus-infected cells. Data depicts the mean with S.D. from three independent experiments. (**H**) Supernatants from DiD-stained infected and uninfected cells were treated with 0.05% Triton X-100.

Our next approach was to stain virus-infected cells *in vitro* such that virus and EVs would egress directly from fluorescently labeled lipid membranes. To our knowledge, this method has been shown to label EVs, but has yet to be shown to label retroviruses. The same panel of dyes as above were tested. Staining on cells was confirmed by fluorescence confocal microscopy (Fig. 5A–E, *right panels*). The population of MLVeGFP virions indirectly labeled with DiD, DiI and FM4-64 was nearly completely resolved from noise by each of these dyes (Fig. 5A,B and C). However in this indirect staining method, virus as well as EV populations were labeled by the dyes, as seen with the EVs released from uninfected cells. DHPE-Rhodamine did not significantly label MLVeGFP (Fig. 5D), while Ceramide appeared to label similarly to the direct staining method, and again, was unable to resolve the virus population from the background (Fig. 5E).

Next we assessed if dye-labeled viruses remained infectious. This could be of use for downstream applications. DiI stained cells were excluded from this assessment, as it is chemically analogous to DiD. The effects of solvents were included as controls. The infectivity of viral supernatants produced in DiD and Ceramide labeled cells was abrogated, while DHPE-Rhodamine and FM4-64 labeled cells produced virus with significantly reduced infectivity (Fig. 5F). Although, virus infectivity may be compromised by the dyes, we also assessed whether treating the infected cells with the dyes affected viral particle release. Indeed, fewer viral particles were released with most of the dyes (Fig. 5G), however this did not account for the total loss of infectivity. To confirm that fluorescent particles were indeed viruses and EVs, samples were treated with 0.05% Triton X-100. The EV population was abolished by detergent treatment, as was eGFP fluorescence emitted by the virus (Fig. 5H).

### Discriminating enveloped viruses from EVs by nucleic acid labeling

We next attempted to discriminate EVs from viruses based on their nucleic acid content. MLVeGFP, similarly to other retroviruses, packages two copies of its ~9Kb single-stranded RNA genome. In addition, retroviruses also package large amounts of cellular mRNAs, tRNAs and non-coding RNAs that represent approximately 50% of total RNA in the virion^55,56^. While EVs are known to also package nucleic acids, both DNA and RNA, the total amount of nucleic acids they package in comparison to retroviruses is unclear^15,57,58^. SYBR Green is a dye that has a strong affinity for nucleic acids, with chemical variants that have been developed with higher affinities and quantum yields for RNA (SYBR II) or DNA (SYBR I). Nucleic acid dyes such as SYBR Green have been shown previously to label virus, however in those studies, it was unclear whether the dye-labeled virus samples were contaminated with EVs^26,29,33^.

**Figure 6:**
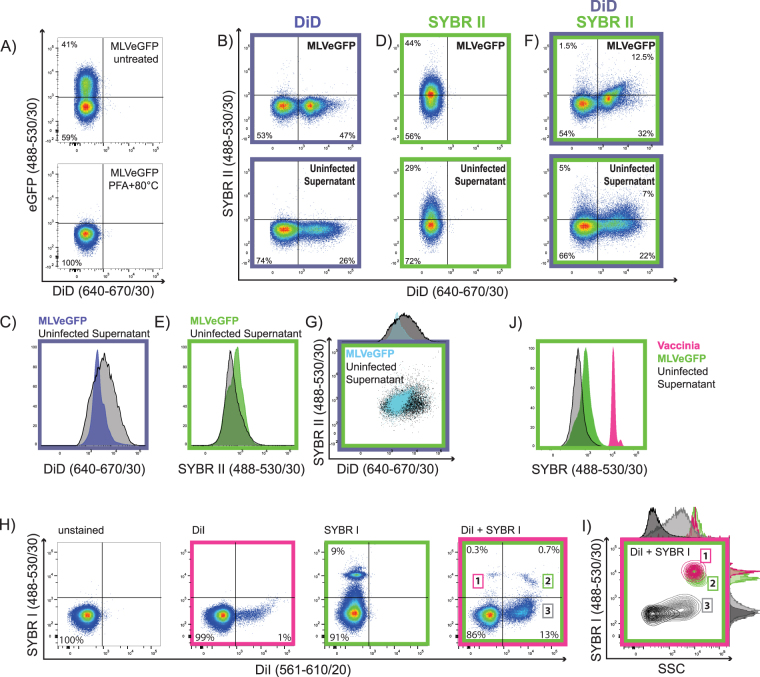
Discrimination of enveloped viruses from EVs by a combination of membrane and nucleic acid dyes. (**A**) MLVeGFP was treated with 2% PFA at 80 °C to fix the virus and permeabilise the envelope and capsid, rendering the content of the particles amenable to staining by nucleic acid dyes. MLVeGFP infected and uninfected cells were stained with DiD, and then particles in the supernatant were analysed directly (**B**), or further stained with SYBR II following fixation and heat treatment (**F**). (**D**) Supernatants from infected and uninfected cells were stained with SYBR II only and analysed. Comparison of fluorescent intensities between supernatants from infected (coloured histograms) and uninfected cells (black histograms): (**C**) DiD stained cells, (**E**) SYBR II stained supernatants, (**G**) histogram and dotplot overlay of dual stained DiD/SYBR II nanoparticles from infected and uninfected cells. Nanoparticles from uninfected cells are depicted in black, and from infected cells in blue. (**H**) Direct labeling of VV with DiI or SYBRI alone, or in combination (fourth panel). (**I**) Relative sizes of nanoparticle populations 1 to 3, displayed as a comparison of SYBRI vs SSC. (**J**) Overlay of SYBR fluorescence intensity from stained MLVeGFP, VV and EV-containing cell supernatant.

To attempt to discriminate EVs from viruses, MLVeGFP was first labeled with the lipid membrane dye DiD followed by SYBR II. The nucleic acid labeling procedure, as described previously^26,29^, requires fixation of the virus followed by staining at 80 °C. This exposure to heat denatured eGFP on the surface of the MLVeGFP virus and it was no longer fluorescent (Fig. 6A), while fixation with 2% paraformaldehyde (PFA) alone had no significant impact on eGFP fluorescence (data not shown). MLVeGFP and uninfected cell supernatants were labeled alone with DiD (indirect) (Fig. 6B and C), or SYBR II (direct) (Fig. 6D and E), or dual labeled (Fig. 6F and G). When the DiD+ population in the MLVeGFP sample was compared with that of the uninfected supernatant control, the two populations exhibited different MFI peaks despite largely overlapping (Fig. 6C). The same was true for the SYBR II-positive and DiD/SYBR II double labeled populations (Fig. 6E and G). Vaccinia virus (VV) is an enveloped DNA virus with a much larger genome of 192 kbp^59^. VV harboring close to 20 times more nucleic acid content was used as a positive control for nucleic acid staining. The combined use of SYBR I with DiI distinctly resolved three populations: DiI-SYBR+ (1), DiI + SYBR+ (2), and DiI + SYBR− (3) (Fig. 6H and I). This was not completely surprising, as it is known that VV can be released as single-membrane and dual-membrane particles, and can also produce genome-deficient viral particles^60–62^. When plotting VV alongside MLVeGFP and EVs, we can clearly discern differences in SYBR staining between these three populations (Fig. 6J). These data indicate that nucleic acid staining is an effective way to distinguish virus with a large genome but not retroviruses from EVs.

## Discussion

Careful optimization of instrument settings is often underappreciated to obtain the best performance from a flow cytometer. In conventional flow cytometers, this optimization is standardized with beads as a reference material. In BD instruments like ours, this is achieved with the automated BD CS&T program and beads, however CS&T target values are optimized for cells. Therefore voltages and laser settings for the analysis of nanoscale particles need to be optimized manually with relevant reference materials, especially since the particles of interest are at the limit of detection of current instruments (90–200 nm). In fact, there exists a large discrepancy in both fluorescence intensity and refractive index (RI) between most calibration beads and biological nanoparticles of interest^63–65^. As such, we chose a fluorescently tagged virus as the standard, which allowed us to optimize our instrument settings on a biologically relevant particle. Alternatively, other groups have been successful at analyzing nanoparticles through conjugation with fluorescent beads^11^. The obvious benefit of these bead conjugates are that they are easily detected on a wide range of flow cytometers, however their multivalent nature may also be a source of bias introduced during data acquisition. Antibody labeling of nanoparticles can in some circumstances induce aggregation^32^. For this reason, we carried out our study on fluorescent viruses having undergone a minimal amount of manipulations that were limited to dye-loading, followed by filtration and dilution. As such, the limits of our method to discriminate between retroviruses and EVs are, for the moment, reserved for research applications where the use of a genetically engineered virus with a fluorescent envelope glycoprotein are possible, such as in cell lines or animal models.

There are advantages and disadvantages in selecting light scatter as the threshold parameter. On the one hand it allows for the detection of all non-fluorescent particles, but on the other hand, with our instrument and software, we see an increase in the overall abort rate. This increased abort rate resulted in a 3-fold reduction in the total eGFP+ events in comparison to fluorescence thresholding (Fig. 2D and E). The choice between SSC and FL for threshold is therefore application-dependant. In an experiment where the enumeration of a population of interest is required, samples should be collected using FL-thresholding; the caveat being that this population must be fluorescently labeled. On the other hand, if the goal of the study is to analyze different populations labeled with mutually exclusive fluorescent marker combinations, then SSC thresholding should be chosen to collect all relevant data, and better appreciate small differences in population relative sizes, clustering and distributions.

When analyzing MLVeGFP viruses that contain approximately 100 Env-eGFP molecules on their surface^43^, we see the greatest gain in fluorescent particle resolution when the power of the 488 nm laser increases from 100 to 200 mW (Fig. 3B and E). Nonetheless, for the detection of antigens at the surface of nanoparticles that are less abundant than Env-eGFP, like for example gp120 on the surface of HIV-1 virions^41,43^, the higher power laser will likely greatly improve signal resolution.

As mentioned earlier, retroviruses share many physical properties with EVs. Yet despite these similarities, MLVs, as a population, are distinct from EVs in that they are more homogeneous in light scattering properties (Fig. 1F). They are also more resistant to the uptake of membrane dyes when directly labeled (Fig. 4), and appear to contain slightly more nucleic acids than the EVs (Fig. 6E). These small differences are important in the context that EVs will contaminate even the purest of retrovirus preparations, as fractionation, filtration, and size exclusion techniques are not entirely effective^5^. Furthermore, given that EVs are known to harbour viral proteins and nucleic acid fragments, and share numerous surface markers, it is most challenging to discriminate them using standard biochemical approaches^5^. If single-particle characterizations are carried out on viruses, it is therefore essential to develop methods to discriminate them from EVs.

In this work, we clearly demonstrate that a fluorescent tag on the retroviral envelope glycoprotein of MLV can serve as an excellent identifier. While providing specificity to detect viral particles, our MLVeGFP virus enabled us to evaluate the efficacy of various membrane dyes and staining methods. We found that retroviruses released in the cell supernatant were generally poorly amenable to staining with membrane dyes, in contrast to EVs (Fig. 4). This may be indicative of small, but significant biochemical differences in the membranous envelope of retroviruses and EVs. However, approximately 8–17% of viruses do appear to have been labeled by the dyes. This does not come as a complete surprise since it has been previously documented that a small fraction of MLVs are released through the endosomal pathway rather than budding at the cell surface and these viral particles might take up dye more efficiently than virus budding at the cell surface^66,67^. This would suggest that these dual-stained retroviruses share even greater biochemical similarities to small EVs, which are mostly comprised of exosomes^17^. In comparison, the indirect staining method may label the viruses non-specifically through uptake of unbound dye present in the cytosol or indirectly through packaging of macromolecules stained by the dye (Fig. 5).

Fluorescent labelling of the virus with dyes had a severe impact on its infectivity (Fig. 5F and G). Even DHPE-Rhodamine, which stained very poorly, had a negative impact on infectivity. Our data indicate that membrane dyes are imposing a defect at egress in the producer cells or are directly affecting the infected cell metabolism (Fig. 5G). It is clear that these staining methods are not suitable for some downstream applications that require infectious virus, and caution should be taken when analyzing viral fitness using chemically stained particles. Similarly, the same caution should be taken when using these dyes with EVs for functional studies.

In a further attempt to differentiate retroviruses and EVs, we investigated if nucleic acid packaging could be a discriminating factor. Though MLVeGFP was unable to be clearly resolved from EVs by dual lipid and nucleic acid staining, it did show superior particle homogeneity and slightly more fluorescence (Fig. 6C,F and G). In contrast, VV which has a much larger physical size (250 × 270 × 360 nm)^68^ and genome than MLVs is clearly resolved by using a fluorescent nucleic acid dye and SSC (Fig. 6I and J).

With technical advancements in flow cytometry it is now possible to visualize nanoparticles in the 90–120 nm size range using NFC. This innovation opens a wide array of new possibilities that result from single particle analysis of viruses and EVs that include the individual profiling of surface antigens, sorting of particles with distinct markers, and even the precise enumeration of particles displaying certain fluorescence or light scattering characteristics. NFC has the potential to bring new understanding to the fields of virology and EV research, as it provides a tool to answer questions that were not previously possible to address.

## Methods

The datasets generated during and/or analysed during the current study are available from the corresponding author on reasonable request.

### Flow cytometer features and specifications

SORP BD LSRFortessa for small particle detection with a PMT for forward scatter detection. Specifications for laser wavelengths and power are as follows: 405 nm– 50 mW, 488 nm–300 mW, 561 nm–50 mW, and 640 nm–40 mW. Acquisition was done with BD FACSDiva version 8.0.1. BD Coherent Connection software was used for laser power adjustments. Samples, unless otherwise indicated, were acquired on the *low* sample pressure setting (at 5 turns on the fine adjustment knob), which equated to a measured flow rate of 20 μl/min. This instrument is run with a BD FACSFlow Supply System (FFSS) for day to day acquisition of cells, however for small particle detection, a dedicated steel sheath tank with a 0.1 μm inline filter was used along with a separate waste tank. This was done because we found the FFSS contributed to excess fluctuations in instrument background noise. Surfactant-free, ultra-filtered, low particle count sheath fluid was used for acquisition (Clearflow Sheath Fluid – Leinco). CS&T was run using the dedicated tank to obtain appropriate laser delays for use with the tank, since there is a difference in pressure between the FFSS and the steel tank. Instrument cleaning procedure prior to acquisition: 10 mins distilled water, 30 min FACSClean (BD Biosciences), 10 min distilled water, 60 min 10% Decon™ Contrad™ 70 Liquid Detergent (Thermo Fisher Scientific), and 10 min distilled water.

### Fluorescence Index calculation

The fluorescence index was calculated as the difference of the median fluorescence intensity of the positive and negative population divided by the standard of deviation of the negative population. Fluorescence index = (MFIpos − MFIneg)/SDneg.

### Data acquisition and analysis

Flow cytometry data was displayed in height for all figures. For small particle analysis, height is the preferred parameter over area. Area is the integrated value of an electronic pulse based on the height and width (time of flight). However, since the particles of interest are very small, the width or time of flight measurements become less precise. This leaves height as the intensity of the signal as the most accurate parameter for analysis of submicron-sized particles.

In our instrument platform, side-scatter was chosen over forward-scattered light detection for the approximation of particle sizes. As approximated by Mie Scatter Theory, the angle of light scatter from a particle in the 100–200 nm size range is such that more light is captured at the side-scatter angle rather than forward, whereas with a cell-sized particle (10 μm) the opposite is true^47,69^. We found that despite having a PMT for FSC detection, resolution in SSC was still superior for our particles of interest. Flow cytometry data was analysed using Flowjo VX (FLOWJO, LLC). GraphPad Prism v6 was used for the generation of graphs (GraphPad Software).

### Fluorescent polystyrene beads

Megamix-Plus SSC fluorescently labeled beads (Biocytex, Marseille, France #7803) with 160 nm, 200 nm, 240 nm, and 500 nm size populations was used. The 500 nm bead population was off scale when run at settings optimized for MLVeGFP resolution.

#### Cells, Plasmids and Viruses

Cell Culture: Human embryonic kidney (HEK) 293 T and mouse embryonic fibroblast NIH 3T3 cells were cultured in DMEM High Glucose Medium (Wisent, St Bruno, Canada), supplemented with 10% Fetal Bovine Serum (FBS, Gibco by Thermo Fischer Scientific, Waltham, MA), 100 U/mL penicillin and 100 µg/mL streptomycin (Wisent, St Bruno, Canada). This media will be referred to as complete media. Propagation was continued at 37 °C in a 5% CO_2_ incubator.

MLVeGFP: Replicative Moloney-MLV, referred to as MLVeGFP throughout this study, was produced from the pMOV-eGFP expression plasmid^70–72^. The eGFP reporter is inserted in frame within the proline-rich region of the viral envelope glycoprotein, is expressed on the exterior surface of the virus, and does not alter infectivity nor ecotropic receptor specificity^70^.

### Production of MLVeGFP from chronically infected cells

Aside from MLVeGFP used in Fig. 1D–H that was produced by plasmid transfection in 293 T cells, MLVeGFP virions were otherwise produced from chronically infected NIH 3T3 cells. For the generation of chronically infected cells, NIH 3T3 cells were infected with MLVeGFP at a very high multiplicity of infection (MOI). In short, 10 mL of MLVeGFP-containing cell supernatant was produced by transfection of 293 T cells in a 10 cm dish for 72 h. The supernatant was cleared through a 0.45 μm filter and was ultra-centrifuged at 100,000 × g for 3 h in a 70Ti rotor at 4 °C. The entire viral pellet was resuspended in DMEM and used to infect NIH 3T3 cells seeded at 500 000 cells/well in a 6-well dish. For virus production, uninfected control cells or chronically infected NIH 3T3 cells were seeded at a density of 5 × 10^5^ cells/well in 6-well plates in complete media. After 18 h, cells were washed three times with 0.1 μm filtered PBS and incubated in 1.5 ml of 0.1 μm filtered, serum-, antibiotic- and phenol red-free DMEM for 3 h. Unless otherwise stated, the cell supernatant was passed through a 0.45 μm acrodisc syringe filter with SuPor (PES) membrane (Pall Corporation, Port Washington, NY, cat. #PN4614) and diluted 100-fold in 0.1 μm ultrafiltered PBS before NFC analysis.

### Production of MLVeGFP by transfection

The plasmid eGFP-C3 (Clontech, Mountain View, CA) was used for cytoplasmic eGFP expression. For the construction of an Env-eGFP expression plasmid, the Env-eGFP gene of pMOV-eGFP was amplified by PCR using the following primers: 5′-GCTAGCGCCGCCACCATGGCGCGTTCAACGCTCTCAAAACC-3′ (forward) and 5′-CTCGAGCTATGGCTCGTACTCTATAGGCTTCAGCTGGTG-3′ (reverse). The amplification product was then inserted between the NheI and XhoI restriction sites of the expression vector pcDNA 3.1 (-) downstream of the CMV promoter.

For virus or EV production, 293 T cells were transfected with pMOV-eGFP, pEnv-eGFP or peGFP-C3. 24 h before transfection, 293 T cells were seeded at a density of 1.25 × 10^5^ cells/well in a 24-well plate in complete DMEM media. For each well, a total of 500 ng of DNA was transfected using GeneJuice (Novagen, EMD Millipore, Billerica, MA) according to manufacturer’s instructions. After 36 h, the cells were washed with PBS and media was changed to 0.1 μm-filtered, serum and phenol red-free DMEM (Wisent), to allow for virus or EV production with minimal contaminants. After 3 h, the cell supernatant was harvested and cleared through a 0.45 μm acrodisc syringe filter with SuPor (PES) membrane (Pall Corporation) unless otherwise specified. For analysis of the effects of microfiltration on viral sample, supernatants were filtered through 0.1 μm (cat. #PN4612), 0.2 μm (cat. #PN4612) or 0.45 μm filters (Pall Corporation). Samples produced from transfections were diluted 1:10 in 0.1 μm-filered PBS prior to analysis by NFC.

### Virus titer calculations

To calculate of the viral titer in transducing units (TU)/mL, known volumes of 0.45 μm-filtered viral supernatant was titrated on 1.0 × 10^5^ NIH 3T3 cells per well in a 12-well plate. Twenty-four hours post-infection, these cells were analyzed by flow cytometry. Infections ranging from 2–30% were assumed to have one productive integration per cell, and used to calculate TU/mL with the following formula: TU/mL = # of infected cells/volume of viral supernatant.

### Viral genome quantifications by ddPCR

Viral RNA genomes were isolated from infected cell supernatants using the QIAamp Viral RNA Mini Kit (Qiagen) following manufacturer’s guidelines. The RNA eluate was then reverse transcribed using the Advanced cDNA Synthesis Kit (Wisent) and analyzed by droplet digital PCR, using the QX200 system (BioRad), for the presence of the eGFP coding sequence. The primers used for this analysis were R279-FWD and R279-REV, as previously described by our group^73^. Data was analyzed using QuantaSoft^TM^ and extrapolated based on the dilutions used for the assay.

### Vaccinia virus stock preparation

VVDD-mCherry stocks were obtained from John C. Bell. Briefly, virus stocks were produced by infecting HeLa cells at a low MOI (0.01). Infected cells were lysed by repeat freeze-thaw cycles (−80/37 °C), cell debris was removed by centrifugation, and virus was clarified through a sucrose cushion at 20,700 × g with a JS-13.1 rotor for 80 min at 4 °C^32,74,75^.

### Nanoparticle tracking analysis and zeta potential measurement

Nanoparticle tracking analysis (NTA) was carried out using the ZetaView PMX110 Multiple Parameter Particle Tracking Analyzer (Particle Metrix, Meerbusch, Germany) in size mode using ZetaView software version 8.02.28. Samples were diluted in PBS to ~10^7^ particles/ml. The system was calibrated using 105 and 500 nm polystyrene beads and then videos were recorded and analyzed at all 11 camera positions with a 2 second video length, a camera frame rate of 30 fps and a temperature of 21 °C. Analysis was performed using Particle Explorer version 1.6.9 (Particle Metrix). Analysis parameters were: segmentation-fixed, centroid estimation-blob, drift compensation-auto, log detection threshold-0.0175, max particle size-1000, min particle size-6.0, segment threshold-18. Results are displayed as the percentage of particles within 25 nm segments and as mean particle size. Zeta potential was measured using the ZetaView PMX 110 Multiple Parameter Particle Tracking Analyzer (Particle Metrix) in zeta potential mode as described previously^76^. All measurements were performed at 21 °C using samples diluted in 0.1x phosphate buffered saline (diluted in double-distilled H_2_O to ensure conductivity of approximately 500 µS/cm. Uninfected supernatants were diluted 1:10 and MLVeGFP was diluted 1:100 to achieve equivalent particle concentrations. Data were analysed using ZetaView software (version 8.02.28). Instrument settings were as follows, sensitivity: 85, frame rate: 30 frames per second, shutter speed: 100. Post-acquisition parameters were set to a minimum brightness of 20, a maximum size of 200 pixels, and a minimum size of 5 pixels. Temperature, conductivity, electrical field, drift, and pH of the diluent were consistent for all samples. The zeta potential of particles released in the supernatent of non-infected cells was −23.2 ± 2.5 mV, and −33.6 ± 2.6 mV for particles in the infected cell supernatant.

#### Lipophilic membrane dyes and nucleic acid labeling

Direct labeling with lipid dyes: Dyes were added directly to undiluted control or MLVeGFP containing supernatant at optimized concentrations indicated below. The dye-labeled control or viral supernatants were diluted 1:10 and 1:100 in 0.1 μm filtered PBS, respectively. These were then filtered with a 0.45 μm pore-size syringe filter prior to acquisition on the cytometer. DiD and DiI solutions were used at 10 µM, while DHPE-Rhodamine, FM 4-64X and BODIPY TR Ceramide were used at 10 µg/mL (all Thermo Fisher Scientific).

Indirect labeling with lipid dyes: Uninfected and infected NIH 3T3 cells were cultured overnight with dye. The following day, cells were washed 3 times with 0.1 μm-filtered PBS to remove excess dye. Following washing, the cells were placed back in the 37 °C incubator with 0.1 μm filtered, serum and phenol red-free media. After 3 h, supernatant was collected, 0.45 μm-filtered (unless otherwise indicated), and analyzed by NFC. As before, control or viral supernatants were diluted 1:10 and 1:100 in 0.1 µm filtered-PBS, respectively. DiD and DiI solutions were used at 25 µM, DHPE-Rhodamine and FM 4-64X were used at 25 µg/mL, and BODIPY TR Ceramide was used at 12.5 µg/mL. DiD and DiI were dissolved in ethanol, while DHPE-Rhodamine, FM 4-64X and BODIPY TR Ceramide were dissolved in methanol. Titrations were performed for both the direct and indirect labeling methods and optimal concentrations were chosen (data not shown).

### Nucleic acid labeling

The protocol for nucleic acid labeling with SYBR Green was adapted from Brussard *et al*.^26,29^. Briefly, supernatants were fixed in 2% methanol-free paraformaldeyde (PFA) solution (Thermo Fisher Scientific, cat. #28906). Virus samples were stained with 1x SYBR Green I (DNA) or SYBR Green II (RNA) at 80 °C for 10 minutes. For dual labeling of MLVeGFP, lipophilic dye was loaded onto the virus by indirect labeling prior to nucleic acid staining. For dual labeling of VV, virus particles were labeled using the direct method post SYBR Green I staining. Samples were diluted and 0.45 μm-filtered after staining for analysis by NFC.

### Fluorescence Microscopy

Uninfected NIH3T3 cells were labeled as described above for the indirect staining method, but scaled down to fit 35 mm dishes (Ibidi, Fitchburg, WI). The Zeiss LSM 880 was used for live imaging confocal microscopy, ImageJ (1.8.0) was used to generate the images.
